# New treatment options for Treatment-Resistant Depression in older adults

**DOI:** 10.1192/j.eurpsy.2026.11411

**Published:** 2026-07-31

**Authors:** M. P. Pando Fernández, P. Martinez Gimeno, M. A. Andreo Vidal, M. Calvo Valcárcel, M. D. L. Á. Guillén Soto, G. Medina Ojeda, A. Rodríguez Campos, L. Rodríguez De Andrés, O. Martin Santiago, M. Rios Vaquero, G. Lorenzo Chapatte, L. Rojas Vazquez, A. Monllor Lazarraga, L. Sobrino Conde, F. J. Gonzalez Zapatero, L. Del Canto Martinez, E. Muñoz Ayuso, I. Larrañaga Gutierrez De Piñeres, D. Garcíamedall Arranz, M. E. Espinosa Muth, P. Anton Machado, S. Díez González

**Affiliations:** Psychiatry, HCUV, Valladolid, Spain

## Abstract

**Introduction:**

Treatment-resistant depression (TRD) is one of the leading causes of global disability (1). This has spurred the search for more effective treatments for treatment-resistant depression (TRD), which is defined as a lack of response to two or more antidepressants and is associated with high morbidity and mortality, including suicide (2).

This has led to the search for more effective alternatives, especially intranasal esketamine. Esketamine has been shown to be more potent than ketamine for the NMDA receptor, allowing for lower doses and reducing dissociative effects. Approved intranasal esketamine has emerged as a promising option for TRD (1), although its high cost and the need for long-term follow-up are disadvantages.

**Objectives:**

To address an emerging treatment such as esketamine in a patient with TRD and consider its implications.

**Methods:**

We conducted a literature review by searching for articles in PubMed.

**Results:**

Treatment with intranasal esketamine is discussed in a 77-year-old female patient with TRD. The patient had undergone several unsuccessful treatment attempts (antidepressants; augmentation strategies with quetiapine and aripiprazole; lithium; electroconvulsive therapy (ECT) was considered but was not feasible due to her implanted cardioverter defibrillator (ICD)).

The patient achieved significant improvement, with a reduction of more than 50% in MADR (Montgomery-Åsberg Depression Rating Scale) and HDRS (Hamilton Depression Rating Scale) scores after 24 weeks.

Clinical diagnosis: F33.2 Major depressive disorder, recurrent severe episode.

Plan:

- Psychopharmacological treatment:
  - Desvenlafaxine 100 mg (1-0-0)
  - Desvenlafaxine 50 mg (1-0-0)
  - Lamotrigine 200 mg (1-0-0)
  - Mirtazapine 30 mg (0-0-1)
  - Clonazepam 0.5 mg (1/4-0-1/2)
- Follow-up with your mental health team in psychiatric consultations.

**Conclusions:**

Intranasal esketamine has been shown to be an effective and well-tolerated treatment with a remarkable rapid antisuicidal effect. Both ketamine and esketamine have been shown to be rapid and effective antisuicidal agents, making them useful in the management of patients in acute crisis (1).

However, their cost and need for long-term use require constant monitoring (2). In the case of our patient, this treatment has been effective for a period of 24 weeks.

Furthermore, although ECT remains a cost-effective option (3), in this case it could not be applied due to the presence of ICD in the patient (4). Preliminary evidence suggests that intravenous ketamine is non-inferior to ECT in its acute efficacy. The efficacy of intranasal esketamine versus ECT in TRD is still being evaluated (4).

**Disclosure of Interest:**

None Declared

